# Supplementary material for: Characterization of Disinfection By-Products Originating from Residual Chlorine-Based Disinfectants in Drinking Water Sources
Source: Toxics. 2024 Nov 9;12(11):808. doi: 10.3390/toxics12110808 (PMC11598810; doi:10.3390/toxics12110808)
Supplement: Supplementary file 1 [file toxics-12-00808-s001.zip › toxics-3271126-supplementary.pdf]

Supplementary information for

Characterization of disinfection by-products originated from residual chlorine-based disinfectants in drinking water sources

Dong-Mei Yang[1], Jia-Le Huang[1], Fen-Li Min[2], Hui-Xian Zhong[1], Jia-Lu Ling[1], Qun Kang[1], Zhao-Hua Li[1], Li-Lian Wen[1*]

1 College of Resource and Environmental Science, Hubei University, Wuhan 430062, China; 202321108012165@stu.hubu.edu.cn (D.-M.Y.); 15972407666@163.com (J.-L.H.); 202221108012325@stu.hubu.edu.cn (H.-X.Z.); 202221108012330@stu.hubu.edu.cn (J.-L.L.); kangqun@hubu.edu.cn (Q.K.); zli@hubu.edu.cn (Z.-H.L.)

2 Hubei Key Laboratory of Environmental and Health Effects of Persistent Toxic Substances, School of Environment and Health, Jianghan University, Wuhan 430056, China; minfenli2024@jhun.edu.cn

* Correspondence: wll@hubu.edu.cn; Tel.: +027-88661699

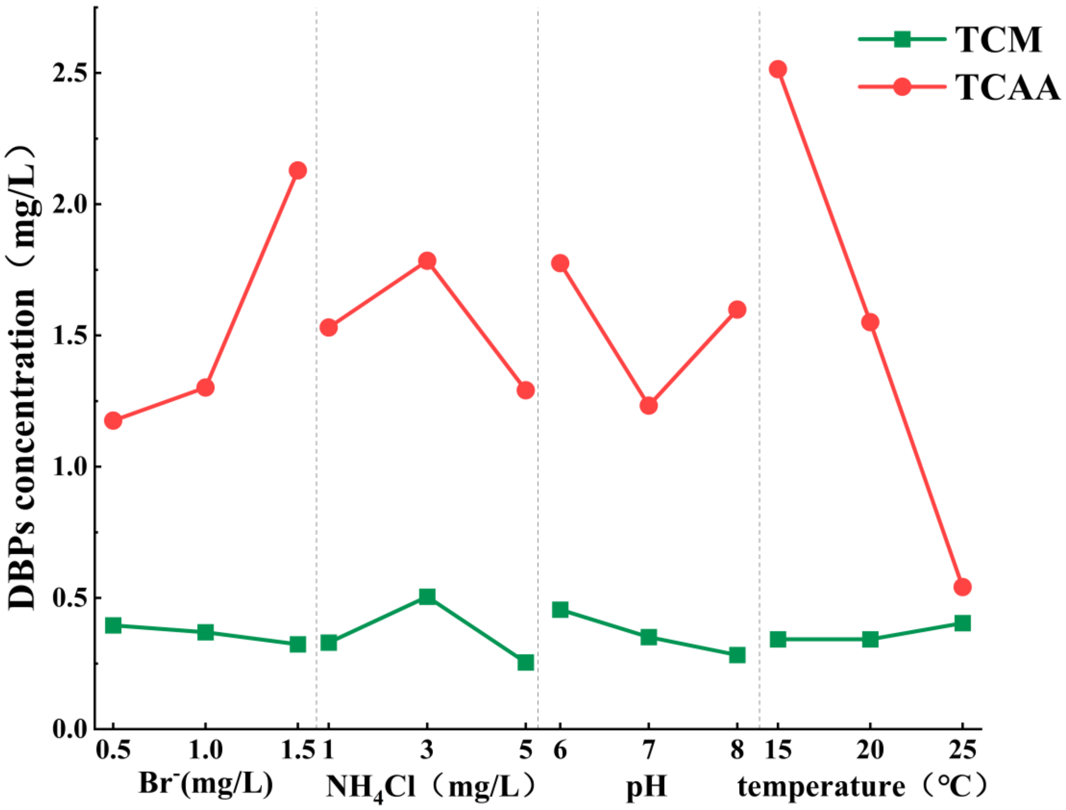

Figure S1. The influence of different factors on disinfection by-products (DBPs) concentration in Han River（trichloromethane (TCM); trichloroacetic acid (TCAA))
